# Economic evaluations of medical devices in paediatrics: a systematic review and a quality appraisal of the literature

**DOI:** 10.1186/s12962-024-00537-0

**Published:** 2024-04-27

**Authors:** Edgar Mascarenhas, Luís Silva Miguel, Mónica D Oliveira, Ricardo M Fernandes

**Affiliations:** 1 Centro de Estudos de Gestão do Instituto Superior Técnico (CEG-IST), Instituto Superior Técnico, Universidade de Lisboa, Avenida Rovisco Pais, 1049-001 Lisboa, Portugal; 2 Centro de Estudos de Medicina Baseada na Evidência, Faculdade de Medicina, Universidade de Lisboa, Lisboa, Portugal; 3 iBB- Institute for Bioengineering and Biosciences and i4HB- Associate Laboratory Institute for Health and Bioeconomy, Instituto Superior Técnico, Universidade de Lisboa, Lisboa, Portugal; 4 Laboratório de Farmacologia e Terapêutica, Instituto de Medicina Molecular, Faculdade de Medicina, Universidade de Lisboa, Lisboa, Portugal; 5 Departmento de Pediatria, Hospital Santa Maria, Centro Hospitalar Universitário Lisboa Norte, Lisboa, Portugal

**Keywords:** Medical devices, Pediatrics, Economic evaluation, Cost-effectiveness, Health technology assessment, Systematic review

## Abstract

**Background:**

Although economic evaluations (EEs) have been increasingly applied to medical devices, little discussion has been conducted on how the different health realities of specific populations may impact the application of methods and the ensuing results. This is particularly relevant for pediatric populations, as most EEs on devices are conducted in adults, with specific aspects related to the uniqueness of child health often being overlooked. This study provides a review of the published EEs on devices used in paediatrics, assessing the quality of reporting, and summarising methodological challenges.

**Methods:**

A systematic literature search was performed to identify peer-reviewed publications on the economic value of devices used in paediatrics in the form of full EEs (comparing both costs and consequences of two or more devices). After the removal of duplicates, article titles and abstracts were screened. The remaining full-text articles were retrieved and assessed for inclusion. In-vitro diagnostic devices were not considered in this review. Study descriptive and methodological characteristics were extracted using a structured template. The Consolidated Health Economic Evaluation Reporting Standards (CHEERS) 2022 checklist was used to assess the quality of reporting. A narrative synthesis of the results was conducted followed by a critical discussion on the main challenges found in the literature.

**Results:**

39 full EEs were eligible for review. Most studies were conducted in high-income countries (67%) and focused on high-risk therapeutic devices (72%). Studies comprised 25 cost-utility analyses, 13 cost-effectiveness analyses and 1 cost-benefit analysis. Most of the studies considered a lifetime horizon (41%) and a health system perspective (36%). Compliance with the CHEERS 2022 items varied among the studies.

**Conclusions:**

Despite the scant body of evidence on EEs focusing on devices in paediatrics results highlight the need to improve the quality of reporting and advance methods that can explicitly incorporate the multiple impacts related to the use of devices with distinct characteristics, as well as consider specific child health realities. The design of innovative participatory approaches and instruments for measuring outcomes meaningful to children and their families should be sought in future research.

**Supplementary Information:**

The online version contains supplementary material available at 10.1186/s12962-024-00537-0.

## Introduction

Advancements in medical technology have led to the development of innovative medical devices that have enabled earlier and more accurate diagnoses, improved treatment outcomes, and helped people live longer and healthier lives. While the medical devices industry has been growing over the last decades [1], this sector has been dominated by the development of devices for adults with few devices being designed and marketed specifically for children [2].

The design and development of medical devices for pediatric populations is challenging namely due to the difficulty in running clinical trials due to highly stringent safety, privacy and ethical requirements - as children are regarded as a vulnerable population - a small market size, making it unattractive to industry and investors [3]. Few devices are submitted yearly for market approval with an explicit indication for pediatric use [4]. In the last decade, only 24% of class III life-saving devices that have been approved by the U.S. Food and Drug Administration (FDA) were for pediatric use– and most of those were designed for children aged 12 and above [5]. Given the lack of pediatric-tailored devices available in the marketplace, pediatricians often extrapolate known adult clinical outcomes and usability data to children, prescribing and using devices devised and approved for adults in pediatric patients, engaging in a so-called ‘off-label’ practice [6]. These procedures are deemed far from optimal, as they may involve considerable risks to the health and well-being of children. Indeed, several adverse events have already been reported [7–10]. To address these issues and compel the industry to advance in pediatric device innovation, specific programs, consortia, and financial incentives have been recently launched in the US [4, 11] and Europe [12]. As a result, new devices for the pediatric population are expected to be developed in the coming years.

Following this, there will be a growing demand for studies and tools to adequately review, synthesise and interpret the evidence on the value of these technologies, in line with providing high-quality and timely information to assist decisions regarding their adoption. By providing a ‘*comparative analysis of both costs and outcomes of alternative health technologies’* (p. 22 [13]),, economic evaluations (EEs) are typically used to inform decisions on the adoption and use of new and emerging health technologies, often within the scope of Health Technology Assessment (HTA) processes.

Any formal and rigorous EE of a given health intervention should reflect the characteristics and context of the population who will benefit from it. When measuring costs and consequences within the pediatric population, additional issues should be considered in the evaluation process, given the specificities of the child’s life and health. These include child’s development trajectory, their dependent status, different patterns of health and disease, and health resource use compared to adults [14–16].

Besides, critical appraisals of EE studies of pediatric drugs and vaccines have detected specific methodological shortcomings, including a poor description of the analytical perspective adopted, a disregard for child-specific and lifelong health outcomes, omission of patient and caregiver productivity costs, and lack of transparency [14, 17, 18].

While these issues may also emerge in evaluating other health interventions, up to our knowledge, no such study has examined the existence of the referred challenges– and perhaps some extra ones– in EE studies of devices used in paediatrics. Thus, there is scope for conducting a review of the published literature, analysing research trends, and mapping methodological issues for informing discussions in light of new advances in the fields of health economics and outcomes research (HEOR) and decision science.

Seizing this research opportunity, this study aims to: (1) identify, review, and characterise published full EEs (comparing both costs and outcomes of two more alternatives) assessing devices used in paediatrics; (2) evaluate their quality of reporting; (3) summarise and critically discuss methodological gaps, with a narrative review, providing recommendations to prompt the generation of more robust and sound EEs focused on devices used in paediatrics. In vitro diagnostic devices (IVDs) are outside the scope of this review, as these are subject to a different EU regulatory framework [19].

Study findings may ultimately contribute to more informed, transparent, and legitimate HTA decisions on the funding, reimbursement, and use of devices for the pediatric population, that fully take into consideration child health realities and unique needs, as well as to a discussion on the relevance for methodological advancements in EE studies for this specific context. To the best of our knowledge, this review is the first specifically focused on EE studies of devices used in paediatrics.

## Methods

The methods for this systematic review were based on the Preferred Reporting Items for Systematic Reviews and Meta-Analysis (PRISMA) 2020 statement [20].

### Information sources

A comprehensive search of the literature (including running the search protocol and collecting the studies) was conducted on 31st March 2022 on the following electronic databases: MEDLINE® In-Process & Other Non-Indexed Citations, MEDLINE® (from 1946) and EMBASE (from 1980), via the Ovid SP interface, Web of Science, Scopus, the Pediatric Economic Database Evaluation (PEDE) [21], the University of York’s Centre for Reviews and Dissemination (CRD) databases, including the HTA and the National Health Service Economic Evaluation (NHS EE) databases. The search was restricted to studies written in English, with no time constraints set in the search period. Reference lists from the retrieved studies were examined to identify additional studies deemed suitable to be included this review.

### Search strategy

A structured search strategy was developed using the PubMed database, with no time limits and restricted to studies written in the English language. The same search strategy was applied to the other databases, with slight changes being performed considering each database-appropriate syntax. Detailed electronic search strategies for each database can be found in the Supplementary Electronic File 1. To achieve adequate sensitivity and identify additional studies, manual searches were also conducted.

### Eligibility criteria

Studies were included if they met the following predetermined inclusion criteria: peer-reviewed journal articles reporting on studies assessing devices in paediatrics in the form of full EEs defined by Drummond et al. as a ‘comparative analysis of alternative courses of action in terms of both their costs and consequences, (p. 22 [13], ) as per stated by the authors or implied by the methods reported; conducted in the pediatric population (≤ 18 years of age). Additionally, the following exclusion criteria were applied: studies assessing health technologies not strictly classified as medical devices, according to the new EU regulation [19]; studies assessing drug-device combination products (e.g. drug eluting stents, metered-dose inhalers); studies assessing sexual/reproductive health devices (e.g. intrauterine devices, vaginal meshes); studies assessing the whole range of in vitro diagnostic (IVD) technologies (e.g. rapid diagnostics tests, enzyme immunoassays); studies assessing digital health technologies (e.g. wellness devices, telemedicine, e-health, mHealth software); studies assessing wearables or fitness tracking devices; studies that reported the use of a device as part of a health intervention, but wherein the main goal was not the assessment of a device (e.g. surgical techniques, newborn screening programs); studies evaluating devices in the adult population; RCTs or other clinical studies providing only efficacy and/or safety evidence or systematic reviews of clinical studies; studies written in a language other than English; study protocols not reporting results, as well as records presented only as abstracts, conference proceedings, case reports, posters, editorials, letters, notes or commentaries; studies with full-text not available. Also, partial EEs– as defined by Drummond and colleagues [13], i.e. ‘studies wherein the costs and consequences of two or more alternative health interventions are not compared’ (e.g. cost analysis, cost description and cost-outcomes description)– were excluded. Systematic literature reviews on EEs and HTA reports on devices were retrieved and analysed to check the completeness of the identified records. Questions regarding the applicability and interpretation of the inclusion and exclusion criteria were discussed with two co-authors (MO and RF).

### Study selection

All the references identified through the searches were imported into reference management software ENDNOTE® X7 (Clarivate Analytics, Philadelphia, PA), and duplicates were removed. An initial screening training set of 10% of the sample was assessed to ensure the appropriateness of exclusion criteria and their consistent interpretation. Titles and abstracts of the retrieved studies were screened by one author (EM) and analysed against the predetermined inclusion and exclusion criteria. Lastly, full-text articles were obtained and thoroughly assessed for eligibility by two reviewers (EM and LSM). Disagreements on whether a specific study should be considered were resolved by a third investigator (MO).

### Data collection and extraction

Data extraction was performed by one author (EM) and validated by a second author (LSM) using a data extraction form designed for this review and implemented in Microsoft Excel. Data collected pertained to: study publication details– the name of the first author, year and journal of publication, article title, first author, country of origin, the country where the study was developed; source of funding and author’s conflict of interest; study descriptive characteristics– a type of the device under assessment and comparators, targeted pediatric age group, study sample size, disease/health condition associated; information on the EE methods employed– a type of EE analysis (e.g. CUA, CEA, CBA), study perspective adopted, time horizon of the study, price year and currency, discount rate for costs, discount rate for outcomes, health outcome measure, direct and/or indirect costs, economic summary measure (e.g. ICER, average cost/QALY), type sensitivity analysis; other relevant information– key results of the study, authors’ conclusions, authors’ stated limitations. The completed Data Extraction Form used in this review can be found in the Electronic Supplementary File 2.

Study perspective refers to the point of view from which the study was conducted [13]. When the perspective was not stated by the authors, we could infer it by the type of costs reported. For instance, if both direct medical costs and indirect costs such as productivity losses were considered, we inferred that a societal perspective was adopted.

### Data analysis and synthesis

Descriptive and methodological characteristics of the included studies were summarized in the form of a qualitative narrative synthesis, complemented with figures and summary tables. Information concerning the device under assessment and comparators, namely the device purpose and risk class were determined according to the new EU regulations for devices (EU 2017/745 and EU 2017/746) [22, 23].

Given the high incidence of CUA studies within our sample, and in light of the challenges concerning the measurement of health-related quality of life (HQROL) and health utilities in children [24, 25], we decided to conduct a distinct analysis for this group of studies, namely examining whether health utilities were prospectively measured, and if so, which method was used, whose health utilities were elicited and who provided the measurement.

### Assessment of quality of reporting

The Consolidated Health Economic Evaluation Reporting Standards (CHEERS) 2022 checklist [26] was applied to evaluate studies’ quality of reporting. This checklist comprises 28 items grouped in six sections: (1) Title; (2) Abstract, (3) Introduction, (4) Methods, (5) Results, (6) Discussion, (7) Other relevant information (Source of Funding, Conflict of Interest). It should be noted that was updated in 2022, with new items being added to the previous checklist. These include for instance aspects related to stakeholder involvement and engagement or the reporting of a health economic analysis plan, among others. One reviewer (EM) completed the CHEERS checklist for each study, indicating “Y” (Yes) when the criteria were met, “N” (No) when they were unfulfilled and “NA” (Not applicable) when they were deemed not required for that type of study. The checklist results were then validated by a second author (LSM). Analyses were conducted to identify (1) the most frequently reported and unreported CHEERS items within our study sample and (2) the studies complying with the majority of the CHEERS items.

## Results

### Search results

**Fig. 1:**
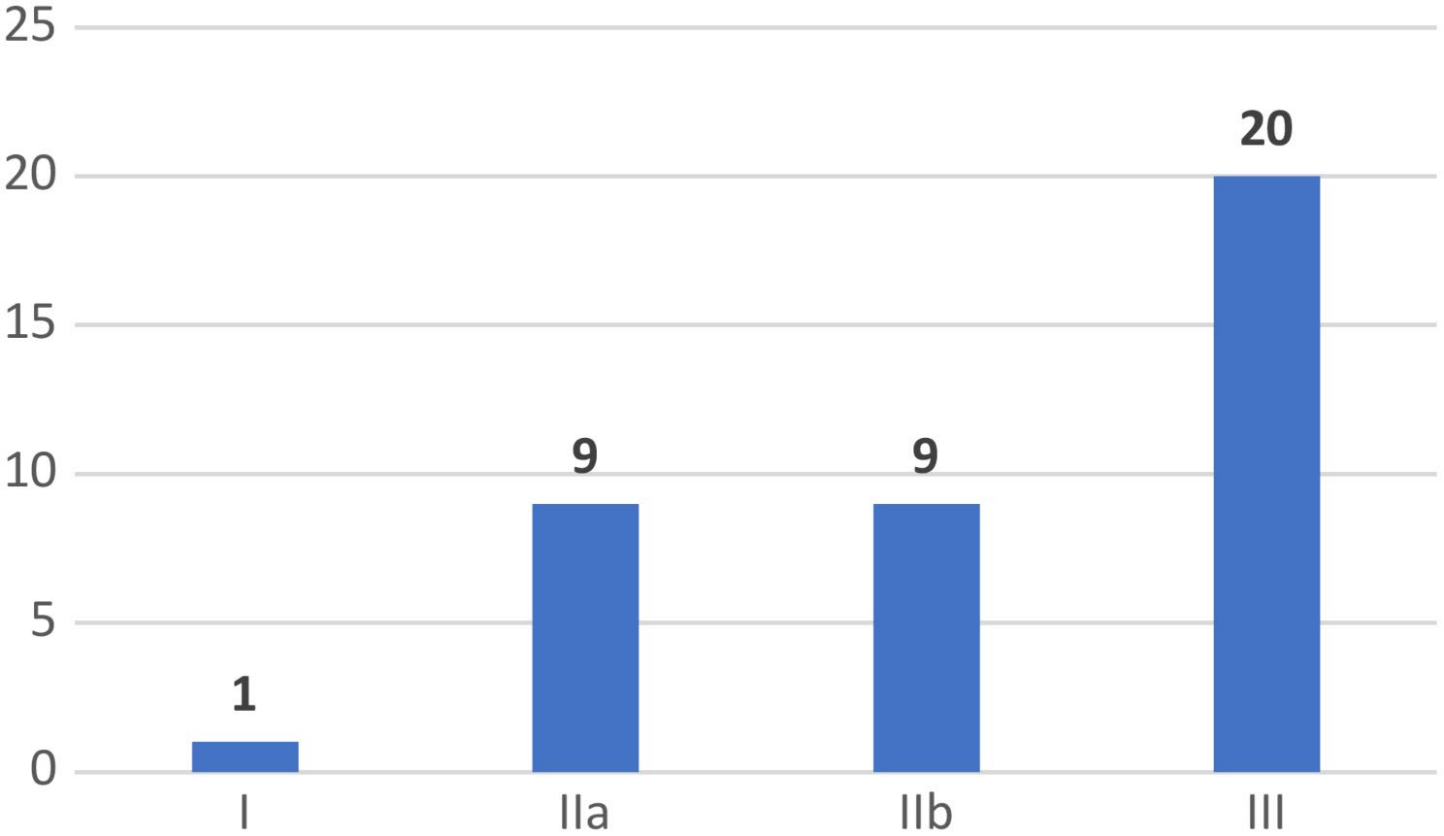
Number of publications according to purpose of the device assessed

Electronic searches yielded 1635 studies, of which 1457 remained after the removal of duplicates. Screening at the title and abstract level resulted in the exclusion of 1201 studies, leaving a total of 256 to be assessed at the full-text level. After full-text screening 32 full EEs were included. The analysis of the reference lists of systematic reviews of EEs and HTA reports led to the inclusion of 7 further studies. Overall, a total of 39 full EEs assessing devices in the pediatric population were included in this review. The results of the study selection process are depicted as a PRISMA flowchart Electronic Supplementary File 3 (See Fig. 1).

### Characteristics of studies

**Fig. 2:**
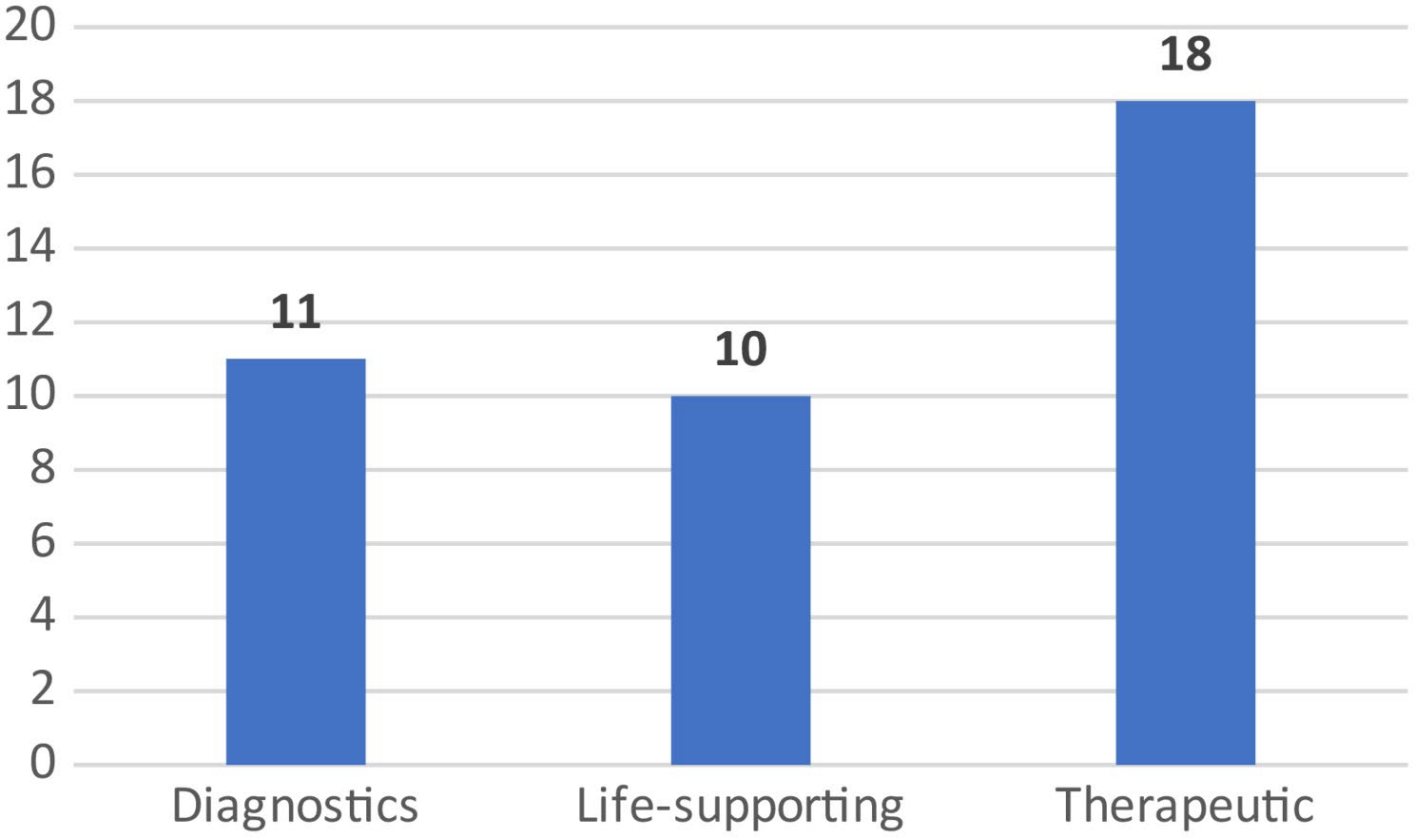
Number of publications according to EU device risk class of the device assessed

**Fig. 3:**
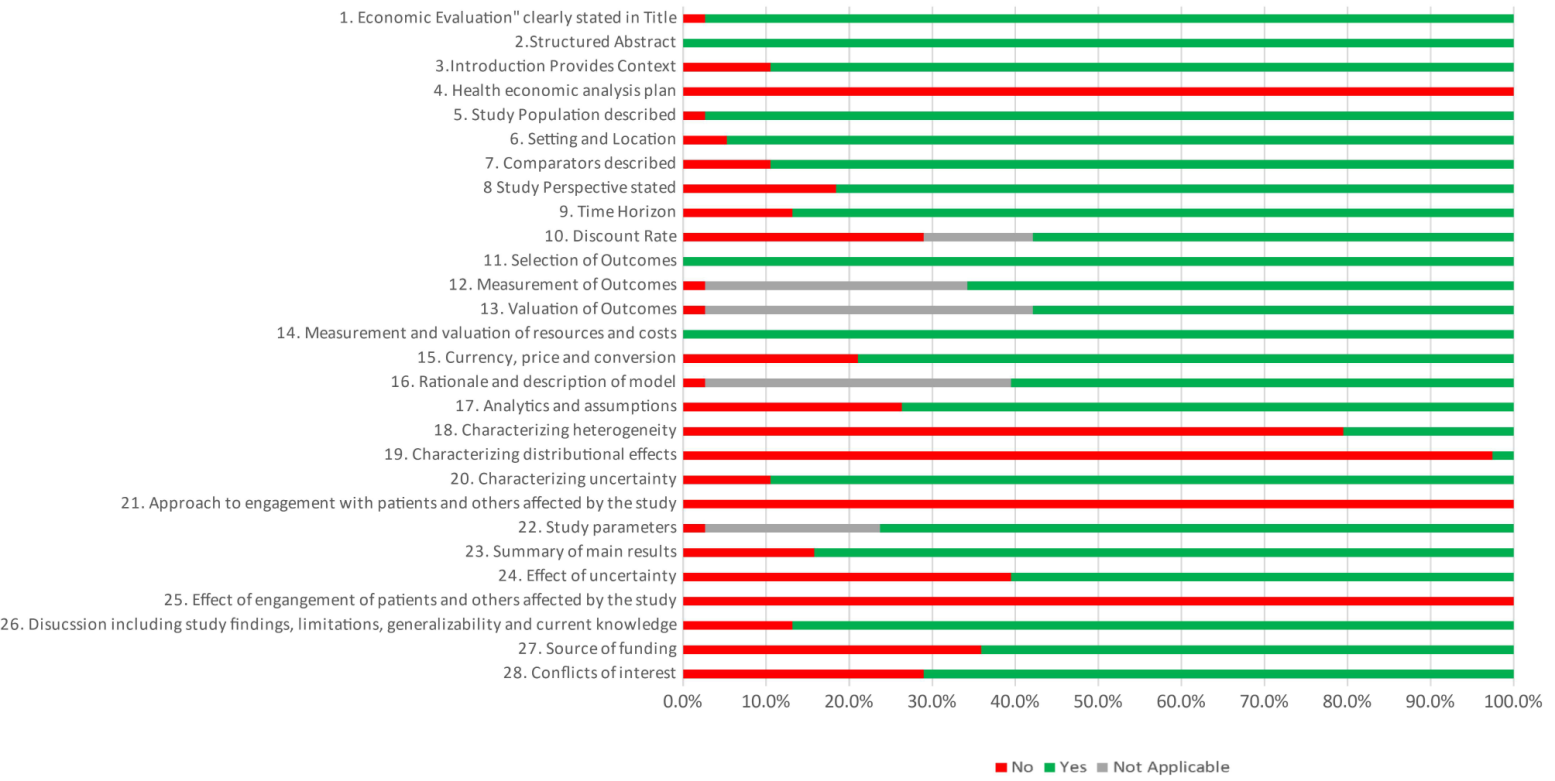
Aggregated results of study quality of reporting by CHEERS item

The 39 full EEs meeting the inclusion criteria were published between 1999 and 2021. Studies are fairly evenly distributed over the years, 64% (*N* = 25) being published in the last 10 years. Nearly 67% (*N* = 26) of the studies were undertaken in high-income countries, 18% (*N* = 7) in upper middle-income countries and 15% (*N* = 6) in low-income or lower-middle-income countries. Most of the studies focused on devices used for therapeutic (*N* = 18, 46%) or life-support purposes (*N* = 10, 26%) (Fig. 2). Cochlear implants being the type of device most frequently reported (*n* = 13, 33%). Higher-risk devices– those classified as EU Class IIb and EU Class III– were assessed in 28 studies (72%) (see Fig. 3). The majority of the EE studies pertained to the medical speciality/area of pediatric otorhinolaryngology (*N* = 13, 33%), followed by neonatology with ten studies (26%) and pediatric cardiology with six (16%). Fourteen studies (36%) did not report the source of funding. Twelve studies (28%) did not report a statement about conflicts of interest. A categorization of each full EE study by type of device, the purpose of the device, EU risk class and targeted pediatric group can be found in Table 1. The summary statistics of the descriptive characteristics of the studies Electronic Supplementary File 3.

**Table 1:** EE Studies by type of device assessed, purpose of device, EU risk class and targeted pediatric group. Medical Device Risk Classification according to the new EU Medical Device Regulation

Author (Publication Year)	Type of device assessed	Purpose of the device	Device EU risk class	Pediatric age group considered in the EE study
Aburahma S (2015)	Vagus Nerve Stimulation device	Therapeutic	III	Infants, children and adolescents
Cheng A (2000)	cochlear implant	Therapeutic	III	Infants and children
O’Neill C (2000)	cochlear implant	Therapeutic	III	Infants and children
Carter R (1999)	cochlear implant	Therapeutic	III	Infants and children
Barton G (2006)	cochlear implant	Therapeutic	III	Infants and children
Foteff C (2016)	cochlear implant	Therapeutic	III	Infants, children and adolescents
Schulze-Gattermann H (2002)	cochlear implant	Therapeutic	III	infants and children
Summerfield A (2010)	bilateral cochlear implant	Therapeutic	III	infants and children
Ahmadi A (2013)	3 types of cardiac duct occluders	Therapeutic	III	Infants, children and adolescents
Brown K (2009)	ECMO device (as a bridge to transplant)	Life-supporting/Therapeutic	IIb	Infants, children and adolescents
El-Saiedi S (2017)	3 types of cardiac duct occluders	Therapeutic	III	infants and children
Heidari S (2017)	auditory brainstem response device	Diagnostics	Iia	neonates
Petrou S (2006)	ECMO	Life-supporting device	IIb	neonates
Mahle W (2008)	Ventricular Assisting Device	Therapeutic	III	neonates, infants, children and adolescents
Campbell K (2007)	Head Computed Tomography (CT)	Diagnostics	IIb	Infants
Chen A (2014)	bubble CPAP device	Life-supporting/Therapeutic	IIb	Neonates
Huang L (2018)	CPAP device	Life-supporting/Therapeutic	IIb	preterms
Santiago Medina L (2001)	diagnostic imaging equipment (MRI, CT, XR)	Diagnostics	IIa and IIb	Neonates
von Keyserlingk K (2011)	wrist splints	Therapeutic	I	infants and children
Santiago Medina L (2002)	3D CT equipment	Diagnostics	IIb	infants and children
Saunders J (2015)	cochlear implant	Therapeutic	III	infants and children
Emmet S (2015)	cochlear implant	Therapeutic	III	infants and children
Pérez-Martin J (2017)	bilateral cochlear implant	Therapeutic	III	Infants
Avanceña A (2021)	implantable ventricular assisting device	Therapeutic	III	adolescents
Cheng L (2019)	bilateral cochlear implant	Therapeutic	III	Infants, children and adolescents
Fang T (2019)	unilateral cochlear implant + contralateral acoustic hearing aid	Diagnostics	Iia	Children and adolescents
Trujillo, D (2019)	pulse oximeter	Life-supporting/Therapeutic	Iia	Neonates
Mowitz, M (2017)	nasal continuous positive pressure device	Diagnostics	Iia	Neonates
Narayen, I (2019)	pulse oximeter	Diagnostics	Iia	Neonates
Peterson, C (2013)	pulse oximetry	Therapeutic	III	Neonates
Qiu, J (2017)	cochlear implant	Diagnostics	Iia	Infants and children
Roberts, T (2012)	pulse oximeter	Diagnostics	Iia	Neonates
Tobe, R (2017)	pulse oximeter	Diagnostics	Iia	Neonates
Mukerji, A (2020)	pulse oximeter	Life supporting/Therapeutic	III	Neonates
Evers, P (2019)	The continuous-flow ventricular assist device	Life supporting/Therapeutic	III	children
Buendía, J. (2021)	High-flow nasal cannula	Life supporting/Therapeutic	IIb	Infants, children and adolescents
Huang, L (2021)	nasal CPAP device	Life-support/Therapeutic	IIb	Neonates
Feingold, B (2010)	Implantable cardioverter-defibrillators	Life-support/Therapeutic	III	Infants, children and adolescents
Neves, L (2021)	wide-field imaging + indirect binocular ophthalmoscopy	Diagnostics	Iia	preterms

CPAP– Continuous Positive Airway Pressure; CT– Computer Tomography; ECMO– Extracorporeal Membrane Oxygenation; MRI– Magnetic Resonance Imaging; VNS– Vagus Nerve Stimulation; XR– X-ray equipment. Targeted pediatric age group according to EU regulations: neonates (preterm and term– 0 to 27 days); infants (28 days to 23 months), children (2 to 11 years) and adolescents (12 to 18 years, inclusive)

### Methodological approaches

Of the 39 studies, 25 were classified as Cost-Utility Analysis (CUA), 13 as Cost-Effectiveness Analysis (CEA) and 1 as Cost-Benefit Analysis (CBA). The majority of studies (*N* = 24, 62%) studies adopted an explicit decision-analytic modelling framework: 13 used Markov models, 9 used decision trees and 2 used a combination of decision tree analysis + Markov models.

Study perspectives varied widely across the studies. In 8 studies the study perspective was not explicitly stated. The perspective most adopted was the health system perspective/third-party payer (*N* = 16). Four studies addressed a governmental perspective, accounting for non-health sector costs, such as education costs. Seven studies adopted a societal perspective, wherein productivity costs borne by the family/patient were included along with the health system and non-health systems expenditures. A hospital perspective was adopted in three studies. Five studies adopted more than one perspective (health system perspective/third-party payer and societal).

Time horizon was reported in 33 (85%) studies. Almost half of the studies (*N* = 16, 41%) adopted a lifetime horizon. Twelve studies adopted a shorter timeframe (≤ 1 year).

Eight studies (21%) did not report any discount rate neither for future costs nor for future health outcomes, five of which had considered a time horizon less or equal to one year. In fifteen studies (38%), costs and outcomes were discounted at the same rate: 3% (*N* = 8), 3.5% (*N* = 3), 5% (*N* = 3) and 6% (*N* = 1).

Of the 25 CUA studies, only 7 measured health utilities as part of the EE, with parents, health care providers or representatives of the general population used as evaluator proxies. Health utilities were obtained directly either by applying direct preference elicitation approaches, namely the Time Trade-Off (TTO) approach and/or the Visual Analog Scale (VAS) in [27, 28] or indirectly by using multi-attribute utility instruments (MAUIs), specifically the Health Utility Index Mark II (HUI-II) in [29], the Health Utility Index Mark III (HUI-III) in [30], the Sintonen HRQOL-15D in [31]. One study [32] used two direct approaches (TTO, VAS) and one indirect approach (HUI-III) to mitigate possible selection or recruitment bias. When health utilities were not measured within the study (18 out of 25), they were obtained from previous literature (*N* = 14) or health care providers opinion (*N* = 2). Surprisingly, none of the studies used generic childhood multi-attribute health status classification instruments, such as the EQ-5D-Y [33] or the Child Health Utility 9D (CHU-9D) [34]. A detailed summary statistics of the methodological characteristics of the studies can be found in Electronic Supplementary File 5.

### Quality of reporting assessment

CHEERS 2022 checklist results for assessing studies’ quality of reporting can be found in the Electronic Supplementary File 6. It can be observed that the CHEERS items less frequently reported were ‘Health economic analysis plan’, ‘study perspective’, ‘discount rate’, ‘Currency: price and conversion’, ‘analytic and assumptions’, ‘characterizing heterogeneity’, ‘characterizing distributional effects’, ‘effects of uncertainty’, ‘effects of engagement with patients’, ‘source of funding’ and ‘conflict of interest’. Figure 3 depicts the aggregated results of study quality of reporting by CHEERS item. In addition, results show that 19 studies fulfilled more than 75% of CHEERS items, of which 14 were published in the last ten years. None of the studies complied with the recently added items to the CHEERS 2022 checklist.

## Discussion

This review aimed at identifying and reviewing existing literature of EE studies on medical devices used in pediatrics, examining their scope and methodological approaches employed, as well as assessing their quality of reporting. On the grounds that systematic reviews are the vehicle for providing information on what is known and where knowledge gaps exist, it can be concluded that there is a great deal yet to be learned on this topic.

The 39 articles meeting the criteria for inclusion show a slight increase in the number of publications in the recent years. Most studies were conducted within North American and European settings– in particular in US and UK– wherein conventional EE methods are well-established and/or formally used for guiding funding and reimbursement decisions of new health technologies [35, 36]. This highlights a clear gap in the literature for evidence that can be applied to low- and middle- income countries (LIMC), wherein the highest burden of pediatric disease lies [37].

Nearly 87% of the studies were published in clinical journals. This high percentage may be related to the lower publication standards for EE studies in clinical journals when compared with health economics journals, as argued in previous research [38]. Also, it may reflect the growing interest of the medical community in the results of the EEs to guide clinical adoption decisions.

The vast majority of studies focused on higher-risk (EU class III) therapeutic devices, in particular active implantable devices, such as cochlear implants. As devices increase in risk and complexity, approval requirements are more rigorous and thus more evidence regarding safety and therapeutic benefit is needed to be submitted to regulatory agencies. As a result, more EE studies are expected to be available for higher-risk devices. Also, while pediatric class IIb and class III devices involve a higher risk and are in general more costly, they also offer substantial therapeutic benefit to children and improvements in quality of life [40].

The perspective taken in an EE study provides a framework for analysis and determines what costs and outcomes to include and how to value them [41]. In our review, the study perspective was stated in more than 50% of the articles. In studies that adopt the societal perspective, ideally all types of costs should be considered, including indirect costs. Yet, of the 6 studies that stated that a societal perspective was used, only 3 included indirect costs (such as parents’ productivity losses). Moreover, because several child health conditions may require a delivery of care in non-medical settings such as the home, schools, and the community, identifying the impacts of resource consumption within these settings provides a more comprehensive assessment of the potential added value delivered by the use of the device, which could be used to inform societal resource allocation decision-making. For instance, cochlear implants in children have shown to result in substantial improvements in children education performance [42] or social participation [43]. Implicit in the discussion above, is the need to evaluate costs and consequences of the use of a device along a time horizon that extends over the child lifetime. Yet, only in nearly half of the included studies was a lifetime horizon considered.

Also, nearly 30% of the studies did not report the potential for any conflict of interest and around 36% of the studies did not declare the funding source. Missing details on these two important pieces of information may introduce bias, thereby hindering the credibility and transparency of the results reported in EE studies.

CUA was the type of EE analysis most often employed, representing 64% of all studies. This may reflect increasing attention to guidelines developed by several national HTA and reimbursement agencies [44–46], commonly favouring the use of CUA for capturing health outcomes in terms of QALYs. Yet, only in 7 of the 25 CUAs, health state utilities were prospectively measured, with proxies being used in all of them. These findings are not surprising, especially in light of the broadly discussed practical and methodological challenges on measuring health utilities in children, particularly in very young children and those with developmental limitations [25, 47–49]. The use of direct utility valuing approaches such as time trade-off (TTO) or standard gamble (SG) for assessing utility weights is not straightforward, as young children are unlikely to fully grasp the concept of time in a way that would allow them to trade risks against the time spent in different health states [50]. In these cases, parents can be used as proxy evaluators, yet their ability to report on more subjective outcomes, such as dimensions of quality of life related to their child’s mood or emotional state, is often disputed [51, 52]. In appreciation of both benefits and limitations of parent proxy and child self-reports, dyad approaches - including both parents and children - for assessing child’s health state have been proposed [53].

Interestingly, none of the studies used generic childhood multi-attribute utility instruments (MAUIs) - such as the EQ-5D-Y [33] or the Child Health Utility 9D (CHU-9D) [34]– as a means of quantifying child health state utilities for the calculation of QALYs within the context of a CUA. The lack of utilization of these instruments may be attributed to various reasons, including concerns about their psychometric performance and validation [54], limited use of these tools in clinical studies to generate data for EEs and HTA reports [54], issues about the appropriateness of existing value set utilities and methodology employed [55], and a concern on whether these tools capture all outcomes relevant to child health and well-being [56].

Within CUA studies, QALYs were the most frequent health outcome measured reported. QALYs provide a means of combining ‘the effects of health interventions on mortality and morbidity into a single index [57], allowing for far-reaching comparisons across multiple disease areas. Despite being routinely used as a standard criterion to evaluate new and innovative health interventions, the QALY approach has been debated in recent years [58–60]. An implicit theoretical assumption of the QALY approach is that QALYs are of equal social value, regardless of the characteristics of the recipient, such as their age or pre- or post-intervention health status. This ‘egalitarian’ nature of QALYs [61] may therefore penalize children in funding decisions, as budgets for childcare are not usually ‘ring-fenced’, with decisions on how to spend scarce health budgets typically spanning both adult and child interventions. Literature has shown that society tends to place a greater weight upon a QALY gained by a child than by an adult [62], fuelling the normative discussion of whether decision makers should consider the QALYs gained by children as deserving a special consideration compared to QALYs gained by adults [63]. In addition, the pediatric population is not homogeneous and is divided in several age groups (neonates, infants, adolescents, etc.), making the estimation of health state utilities very challenging.

Recent studies [64–66] have forewarned that, compared to other health technologies, medical devices require a more flexible and tailored approach to economic evaluation. Several methodological challenges have been identified and include the existence of a ‘learning curve effect’, broad organisational impacts, the continuous improvement and rapid incremental innovation; multiple cost components or high variations in pricing [67]. None of the studies measured the extent to which these devices’ distinctive characteristics had an impact on the cost-effectiveness results, let alone acknowledged as critical aspects to explicitly incorporate within an EE. Indeed, it could be suggested that these considerations were omitted in the reviewed EE studies, because of the presumed low impact on the ICER result. Yet, this would require a quantification of their impact, which was not provided within these studies. Although not all device specific characteristics may be applicable to every type of device [68], it is worth to discuss their pertinence in the context of pediatric devices and how can they be incorporated in future EEs.

Learning effects are commonly observed in surgical interventions of therapeutic implantable devices [69]. For instance, Varabyova et al. [70] measured the effect of device operator learning on two health outcomes: in-hospital mortality and hospital length of stay (LOS) for adult patients undergoing endovascular aneurysm repair (EVAR). The authors used Bayesian hierarchical regression models with random effects at the hospital level to estimate the learning curves. A substantial learning effect in EVAR in terms of the improvement in both mortality and LOS was observed. In the same study, a general methodological framework on how to integrate the effect of learning into an EE was proposed. This approach could be extended to the economic assessment of implantable devices routinely used in pediatrics, including cochlear implants, cardiac occulders, pacemakers, implantable cardioverter defibrillators (ICDs).

Moreover, most of devices are also often subject to incremental innovation [71] - product modifications aimed at easing the delivery of a procedure or convenience of use and/or increasing its performance in terms of health outcomes - examples being software upgrades or enhanced battery life. As incremental innovation is a continuous process [72], it makes it difficult to determine when the ‘improvements’ would actually take place and whether this would have an impact on the costs and/or outcomes of a given technology. In this case, consideration of post-market real world data could be useful to adjust initial assumptions with regard to cost-effectiveness results.

Organizational impact refers to the impact of the introduction of new devices mainly at the hospital or health provider level [73]. Heavy and large medical equipment, such as those used in diagnostic imaging technologies, such as computerized tomography [6] or magnetic resonance imaging (MRI) scanners, are associated with a substantial initial investment. Additionally, life-support devices, such as extracorporeal membrane oxygenation (ECMO) machines or continuous positive airway pressure (CPAP) devices, require special training for health care professionals and a redefinition of care pathways may be needed. To quantify potential impacts associated with the integration of a new equipment at a hospital level, modelling techniques, such as discrete event simulation (DES) could be used.

At a broader level, there is a need to go beyond the conventional EE approaches that only consider effectiveness or a health outcome as the unique measure for quantifying the value of a health technology. It has been recognized that these approaches do not to capture in a simultaneous and structured way all the relevant dimensions of value, such as wider innovation, patient convenience, equity aspects or the socioeconomic impact [74]. In the scope of child health, other aspects are deemed relevant to incorporate in an EE study, such as changes in parent/caregiver productivity and earnings, child educational attainment and future employment [75]. Also of importance is the need to collect the views and perspectives of those of are directly and indirectly impacted by the use of the technology. Increased stakeholder involvement in child health EE studies, including the children themselves, their parents and close family relatives can improve the real-world value and applicability of EE studies on child interventions. In response to the issues raised above, Multiple Criteria Decision Analysis (MCDA) has emerged as a reliable alternative or supplementary approach to traditional EE methods, with several studies supporting its use within HTA processes [76–78]. Framed within the field of decision analysis, MCDA offers a structured approach to understand the relative value of health technologies, by considering an explicit set of criteria under a fully transparent process, incorporating a wide range of stakeholder views and preferences [79]. Although MCDA holds promise in the context of HTA, a recent study [81] showed that there is still a need to develop research and clearer methodological guidelines to promote quality and scientific rigor in the use of MCDA in HTA.

This review presents a number of limitations that are worth to be discussed. First, this study only included published literature in peer-reviewed scientific journals and did not consider grey literature, which may have introduced some evidence selection bias. Second, despite having applied comprehensive search strategies to maximize search sensitivity we cannot exclude the possibility of having missed some potentially relevant studies, in particular given the scarcity of search filters focused on medical device interventions. Restricting our search to English-written publications may also have an impact on the results. Third, we did not consider for inclusion in this review partial EEs, which could have enabled a far-reaching understanding of the types of costs, outcomes and risks that are considered when evaluating devices in pediatrics. Yet, as partial EEs only describe the costs and consequences of alternatives alone, without comparing between them, these types of studies are seldom deemed appropriate for informing priority-setting and resource allocation decisions. Fourth, we used the CHEERS 2022 checklist to assess for the quality of reporting of the included studies. Despite being increasingly used to produce transparent and standardized quality assessment of EE studies, checklists are not without limitations [81]. As previous research has shown, completing checklists for appraising the quality of EEs can be difficult and highly subjective [82], potentially hindering the interpretation of the results obtained. Thus, it is possible that we may have underestimated or overestimated the reporting quality of the studies included in this review. Moreover, as the CHEERS checklist does not provide an official scoring or grading scheme (for instance, set cut-off points or a definition of categories for rating the quality of reporting), summing the number of criteria achieved was assumed a suitable approach to differentiate the reporting quality of the studies.

The EE studies included in our review may also be subject publication and outcome reporting biases, which can affect the validity of the study findings. Publication bias occurs when the publishing of research findings is influenced by the direction or strength of the evidence [83] and outcome reporting bias, on the other hand, occurs when only a subset of outcomes, typically those most favourable, are reported [84]. None of the studies included in this review mentioned or assessed the potential occurrence of these biases Given the unique challenges that pediatric populations present to controlled clinical trials, such as small sample sizes, reluctance to test interventions on children, difficulties obtaining informed consent, and limited funding for pediatric research clinical studies and EE studies conducted in pediatric populations may be particularly susceptible to publication and outcome reporting biases [85]. To minimize the potential for such biases, measures such as mandatory registration of all pediatric clinical studies at inception (including non-trial research), increased access to additional documentation on study protocols and methodology, publication of negative or non-significant results, or the use of publication checklists to ensure reporting standards have been met may be implemented [86].

## Implications for research and policy

Building on the findings of this review, a number of opportunities can be drawn to improve current approaches to the economic evaluation of devices in pediatrics. There is an evident need go beyond conventional EE approaches and to advance in sound methods that can capture in a structured way all the relevant dimensions of value related to child health. To this end, more research should be developed in understanding how to effectively engage the children themselves and their parents, through innovative participatory approaches and novel preference elicitation methods, resulting in a better incorporation of child health realities and experiences. Also, efforts should be directed to advance in the understanding of device-specific characteristics and how these can be included into formal economic assessments. Moreover, it would be important to create specific methodological and reporting guidelines for conducting EEs on devices for pediatrics, and that could consider for different HTA decision contexts. Enhanced inclusion of experts in child health could provide a more meaningful assessment, especially in light of the lack of high-quality clinical evidence. On the policy domain, HTA agencies and organizations should enforce strict requirements on the submission of EE studies, namely on specifying how particular methodological challenges were dealt with, thereby increasing transparency and legitimacy in HTA decision-making processes.

## Conclusion

Increasingly EE studies are used in the context of the allocation of scarce healthcare resources to inform decisions on the funding, reimbursement, and use of new and emerging health technologies. Determining the economic value of devices in the pediatric population is not a straightforward, as it comprises various challenges, from the ones posed by device-distinctive characteristics and impacts on users and organizations, to the ones associated with applying conventional economic evaluation methods, which are not well calibrated for children. Lack of adequate consideration of these aspects may affect the validity of reported cost-effectiveness estimates, which can lead to suboptimal decisions on the adoption of devices for children. Our study shows that more effort is needed to advance on methods that can adequately deal with these challenges so that allocation decisions maximize social welfare and achieve efficiency within health systems worldwide, where all members of society, particularly the more vulnerable ones can benefit.

### Electronic supplementary material

Below is the link to the electronic supplementary material.

Supplementary Material 1

Supplementary Material 2

Supplementary Material 3

Supplementary Material 4

Supplementary Material 5

Supplementary Material 6

## Data Availability

All data generated or analysed during this study are included in this published article and its supplementary information files.
