# Supplementary material for: Economic evaluations of medical devices in paediatrics: a systematic review and a quality appraisal of the literature
Source: Cost Eff Resour Alloc. 2024 Apr 27;22:33. doi: 10.1186/s12962-024-00537-0 (PMC11056067; doi:10.1186/s12962-024-00537-0)
Supplement: Supplementary file 1 — Supplementary Material 1 [file 12962_2024_537_MOESM1_ESM.pdf]

Electronic Supplementary File 2 - Search Strategies

Journal: Cost Effectiveness and Resource Allocation

Article title: Economic evaluations of medical devices in paediatrics: a systematic review and quality appraisal of the literature.

Authors: *Edgar Mascarenhas[1], Luís Silva Miguel[2], Mónica Oliveira[1], Ricardo Fernandes[3,4]

Affiliations:

[1]Centro de Estudos de Gestão do Instituto Superior Técnico (CEG-IST), Instituto Superior Técnico, Universidade de Lisboa, Lisboa, Portugal.

[2]Centro de Estudos de Medicina Baseada na Evidência, Faculdade de Medicina, Universidade de Lisboa, Lisboa, Portugal.

[3]Laboratório de Farmacologia Clínica e Terapêutica, Instituto de Medicina Molecular, Faculdade de Medicina, Universidade de Lisboa, Lisboa, Portugal.

[4]Departamento de Pediatria, Hospital Santa Maria, Centro Hospitalar Universitário Lisboa Norte, Lisboa, Portugal.

Corresponding author:

*Edgar Mascarenhas (ORCID: 0000-0002-5375-0644)

edgar.mascarenhas@tecnico.ulisboa.pt

Centro de Estudos de Gestão do Instituto Superior Técnico (CEG-IST), Instituto Superior Técnico, Universidade de Lisboa, Avenida Rovisco Pais, 1049-001 Lisboa, Portugal.

Search Strategies

Embase and Medline (via Ovid)

1 (pediatric* or paediatric* or child* or adolescent*OR toddler* or teen* or perinate* or neonate* or newborn* or infant* or bab* or young*).ab,kw,ti.
2 ((device* or equipment* or technolog*) adj3 (screening or diagnostic* or therapheutic* or medical or clinical)).ab,kw,ti.
3 (HTA or assess* or evaluat* or select* or apprais* or analys* or analyz* or stud* or review*) ti. (8914880)
4 (value or cost* or economic* or benefit* or utilit* or gain* or effect* or efficac* or risk* or harm* or hazard* or safe* or impact* or utilization or utilisation or "use" or outcome* or consequence*).ab,kw,ti.
5 1 and 2 and 3 and 4
6 remove duplicates from 5
7 limit 6 to English language

Web of Science

TS = ((pediatric* OR paediatric* OR child* OR adolescent* OR toddler* OR teen* OR perinate* OR neonate* OR newborn* OR infant* OR bab* OR young*) AND ((clinical OR medical OR screening OR diagnostic* OR therapeutic*) NEAR/3 (device* OR equipment* OR technolog*)) AND TI = (HTA OR assess* OR evaluat* OR select* OR apprais* OR analys* OR analyz* OR stud* OR review) AND TS = (value OR cost* OR economic* OR benefit* OR utilit* OR gain* OR effect* OR efficac* OR risk* OR harm* OR hazard* OR safe* OR impact* OR utilization OR utilisation OR "use" OR outcome* OR consequence*)

Scopus

TITLE-ABS-KEY (pediatric* OR paediatric* OR child* OR adolescent* OR toddler* OR teen* OR perinate* OR neonate* OR newborn* OR infant* OR bab* OR young*) AND TITLE-ABS-KEY ((screening OR diagnostic* OR therapeutic* OR medical OR clinical) PRE/3 (device* OR equipment* OR technolog*)) AND TITLE (HTA OR assess* OR evaluat* OR select* OR apprais* OR analys* OR analyz* OR stud* OR review) AND TITLE-ABS-KEY (value OR cost* OR economic* OR benefit* OR utilit* OR gain* OR effect* OR efficac* OR risk* OR harm* OR hazard* OR safe* OR impact* OR utilization OR utilisation OR "use" OR outcome* OR consequence*)

PEDE Database

Search on PEDE Database has only 3 field boxes and does not allow either the use of Boolean Operators nor combinations of search terms across each field box. By default, searches include all years (until Dec 2021) and all paediatric age subgroups. Thus, we introduced the word 'device' as a search term to retrieve potentially relevant records.

NHS EE, HTA Databases (Centre of Reviews and Dissemination, University of York)

(pediatric* or paediatric* or child* or adolescent* OR toddler* or teen* or perinate* or neonate* or newborn* or infant* or bab* or young*) AND ((device* or equipment* or technolog*) adj3 (screening or diagnostic* or therapeutic* or medical or clinical)).ab,kw,ti.
