# Supplementary material for: Economic evaluations of medical devices in paediatrics: a systematic review and a quality appraisal of the literature
Source: Cost Eff Resour Alloc. 2024 Apr 27;22:33. doi: 10.1186/s12962-024-00537-0 (PMC11056067; doi:10.1186/s12962-024-00537-0)

Electronic Supplementary File 3 – PRISMA Flowchart

Journal: Cost Effectiveness and Resource Allocation

Article title: Economic evaluations of medical devices in paediatrics: a systematic review and quality appraisal of the literature.

Authors: *Edgar Mascarenhas[1], Luís Silva Miguel[2], Mónica Oliveira[1], Ricardo Fernandes[3,4]

Affiliations:

[1]*Centro de Estudos de Gestão do Instituto Superior Técnico (CEG-IST), Instituto Superior Técnico, Universidade de Lisboa, Lisboa, Portugal.*

[2]*Centro de Estudos de Medicina Baseada na Evidência, Faculdade de Medicina, Universidade de Lisboa, Lisboa, Portugal.*

[3]*Laboratório de Farmacologia Clínica e Terapêutica, Instituto de Medicina Molecular, Faculdade de Medicina, Universidade de Lisboa, Lisboa, Portugal.*

[4]*Departamento de Pediatria, Hospital Santa Maria, Centro Hospitalar Universitário Lisboa Norte, Lisboa, Portugal.*

Corresponding author:

*Edgar Mascarenhas (ORCID: 0000-0002-5375-0644)

edgar.mascarenhas@tecnico.ulisboa.pt

Centro de Estudos de Gestão do Instituto Superior Técnico (CEG-IST), Instituto Superior Técnico, Universidade de Lisboa, Avenida Rovisco Pais, 1049-001 Lisboa, Portugal.

PRISMA Flowchart

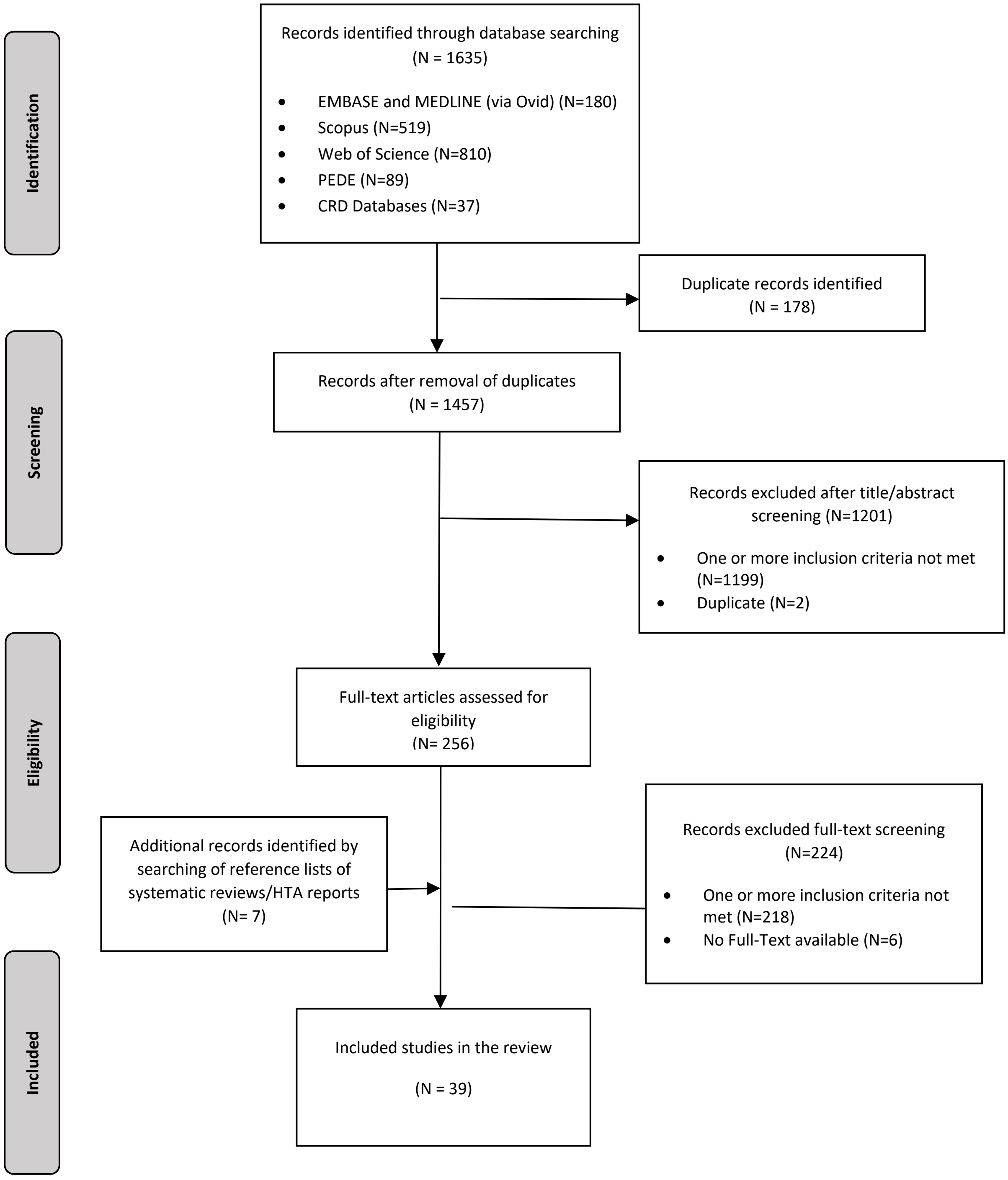

Supplement: Supplementary file 2 — Supplementary Material 2 [file 12962_2024_537_MOESM2_ESM.pdf]
