# Supplementary material for: Economic evaluations of medical devices in paediatrics: a systematic review and a quality appraisal of the literature
Source: Cost Eff Resour Alloc. 2024 Apr 27;22:33. doi: 10.1186/s12962-024-00537-0 (PMC11056067; doi:10.1186/s12962-024-00537-0)
Supplement: Supplementary file 4 — Supplementary Material 4 [file 12962_2024_537_MOESM4_ESM.docx]

**Electronic Supplementary File 4 - Descriptive characteristics of the full EE studies assessing devices used in paediatrics**

**Journal**: Cost Effectiveness and Resource Allocation

**Article title**: Economic evaluations of medical devices in paediatrics: a systematic review and quality appraisal of the literature.

**Authors:** *Edgar Mascarenhas^1^, Luís Silva Miguel^2^, Mónica Oliveira^1,3^, Ricardo Fernandes^4,5^

**Affiliations:**

*^1^Centro de Estudos de Gestão do Instituto Superior Técnico (CEG-IST), Instituto Superior Técnico, Universidade de Lisboa, Lisboa, Portugal.*

*^2^Centro de Estudos de Medicina Baseada na Evidência, Faculdade de Medicina, Universidade de Lisboa, Lisboa, Portugal.*

*^3^ iBB- Institute for Bioengineering and Biosciences and i4HB- Associate Laboratory Institute for Health and Bioeconomy, Instituto Superior Técnico, Universidade de Lisboa, Portugal*

*^4^ Laboratório de Farmacologia Clínica e Terapêutica, Instituto de Medicina Molecular, Faculdade de Medicina, Universidade de Lisboa, Lisboa, Portugal.*

*^5^ Departamento de Pediatria, Hospital Santa Maria, Centro Hospitalar Universitário Lisboa Norte, Lisboa, Portugal.*

**Corresponding author:**

*Edgar Mascarenhas (ORCID: 0000-0002-5375-0644)

edgar.mascarenhas@tecnico.ulisboa.pt

*Centro de Estudos de Gestão do Instituto Superior Técnico (CEG-IST), Instituto Superior Técnico, Universidade de Lisboa, Avenida Rovisco Pais, 1049-001 Lisboa, Portugal.*

**Table** – Descriptive characteristics of the full EEs assessing devices used in paediatrics.

| **Study Characteristics** | **(n=39)** | |
| --- | --- | --- |
|  | ***n*** | ***%*** |
| **Year of Publication** |  |  |
| 1999-2002 | 4 | 10.3 |
| 2003-2006 | 4 | 10.3 |
| 2007-2010 | 5 | 12.8 |
| 2011-2014 | 5 | 12.8 |
| 2015-2018 | 11 | 28.2 |
| 2019-2022 | 10 | 25.6 |
| **Scope of Publication Source** |  |  |
| Health Economics/HTA journal | 4 | 10.3 |
| Clinical/Medicine journal, not paediatric-specific | 19 | 48.7 |
| Paediatric-specific journal | 15 | 38.5 |
| General scope | 1 | 2.6 |
| **Affiliation of the first author (by Continent)** |  |  |
| Australia | 4 | 10.26 |
| Africa | 1 | 2.6 |
| Asia | 7 | 17.9 |
| Europe | 9 | 23.1 |
| North America | 15 | 38.5 |
| South America | 3 | 7.7 |
| **Region of Study (by Continent)** |  |  |
| Australia | 4 | 8.2 |
| Africa | 3 | 6.1 |
| Asia | 7 | 14.3 |
| Europe | 9 | 18.3 |
| North America | 11 | 22.4 |
| South America | 3 | 8.2 |
| Multi-continent | 1 | 2.0 |
| **Purpose of the device** |  |  |
| Diagnostic | 11 | 28.2 |
| Life supporting/Therapeutic | 10 | 25.6 |
| Therapeutic | 18 | 46.2 |
| **Device Risk Class** ^a^ |  |  |
| I | 1 | 2.6 |
| IIa | 9 | 23.1 |
| IIb | 9 | 23.1 |
| III | 20 | 51.3 |
| **Paediatric sub-speciality areas** |  |  |
| Paediatric Otorhinolaryngology | 13 | 33.3 |
| Neonatology | 10 | 25.6 |
| Paediatric Cardiology | 6 | 15.8 |
| Paediatric Pulmonology | 3 | 7.7 |
| Paediatric Neurology | 3 | 7.7 |
| Paediatric Critical Care Medicine | 1 | 2.6 |
| Paediatric Ophthalmology | 1 | 2.6 |
| Paediatric Emergency Medicine | 1 | 2.6 |
| Paediatric Orthopaedics | 1 | 2.6 |

^a^ Medical Device Risk Classification according to the new EU Medical Device Regulation. Class I (low risk), Class IIa (medium risk), Class IIb (medium/high risk) and ending with Class III (high risk).
