# Supplementary material for: Economic evaluations of medical devices in paediatrics: a systematic review and a quality appraisal of the literature
Source: Cost Eff Resour Alloc. 2024 Apr 27;22:33. doi: 10.1186/s12962-024-00537-0 (PMC11056067; doi:10.1186/s12962-024-00537-0)
Supplement: Supplementary file 5 — Supplementary Material 5 [file 12962_2024_537_MOESM5_ESM.docx]

**Electronic Supplementary File 5 - Methodological characteristics of the full EE studies assessing devices used in paediatrics**

**Journal**: Cost Effectiveness and Resource Allocation

**Article title**: Economic evaluations of medical devices in paediatrics: a systematic review and quality appraisal of the literature.

**Authors:** *Edgar Mascarenhas^1^, Luís Silva Miguel^2^, Mónica Oliveira^1^, Ricardo Fernandes^3,4^

**Affiliations:**

*^1^Centro de Estudos de Gestão do Instituto Superior Técnico (CEG-IST), Instituto Superior Técnico, Universidade de Lisboa, Lisboa, Portugal.*

*^2^Centro de Estudos de Medicina Baseada na Evidência, Faculdade de Medicina, Universidade de Lisboa, Lisboa, Portugal.*

*^3^Laboratório de Farmacologia Clínica e Terapêutica, Instituto de Medicina Molecular, Faculdade de Medicina, Universidade de Lisboa, Lisboa, Portugal.*

*^4^Departamento de Pediatria, Hospital Santa Maria, Centro Hospitalar Universitário Lisboa Norte, Lisboa, Portugal.*

**Corresponding author:**

*Edgar Mascarenhas (ORCID: 0000-0002-5375-0644)

edgar.mascarenhas@tecnico.ulisboa.pt

*Centro de Estudos de Gestão do Instituto Superior Técnico (CEG-IST), Instituto Superior Técnico, Universidade de Lisboa, Avenida Rovisco Pais, 1049-001 Lisboa, Portugal.*

**Table** – Methodological characteristics of full EEs assessing devices used in paediatrics.

| **Methodological Characteristics** | **(N=39)** | |
| --- | --- | --- |
|  | ***n*** | ***%*** |
| **Type of EE analysis** |  |  |
| CEA | 13 | 33.3 |
| CUA | 25 | 64.1 |
| CBA | 1 | 2.6 |
| **Decision-analytic framework** |  |  |
| No | 12 | 30.8 |
| Yes | 27 | 69.2 |
| Markov Model | 12 |  |
| Decision Tree | 9 |  |
| Markov model + Decision Tree | 2 |  |
| Unspecified modelling | 4 |  |
| **Study Perspective (according to author)** |  |  |
| Health Care System/Third-party Payer | 14 | 35.9 |
| Governmental | 1 | 2.6 |
| Hospital | 1 | 2.6 |
| Societal alone | 9 | 23.1 |
| Societal + Health Care System/Third-party Payer | 3 | 7.7 |
| Health Care System + Governmental | 1 | 2.6 |
| Health Care payer + Patient | 1 | 2.6 |
| Not stated | 9 | 23.1 |
| **If authors stated Societal perspective, was it?** |  |  |
| Yes | 10 | 83.3 (10/12) |
| No | 2 | 16.7 (2/12) |
| **Time Horizon** |  |  |
| 1 year or less | 8 | 20.5 |
| > 1 – 10 years | 3 | 7.7 |
| > 10 years and <20 years | 5 | 12.8 |
| Child Lifetime | 14 | 35.9 |
| Multiple time horizons (including child' lifetime) | 2 | 5.1 |
| Multiple time horizons (not including child lifetime) | 2 | 5.1 |
| Not specified | 5 | 12.8 |
| **Discount Rate** |  |  |
| Both Costs and Outcomes Rate are specified and have the same value | 19 | 48.7 |
| Both Costs and Outcomes Rate are specified and have different values | 0 | 0.0 |
| Only Cost Discount Rate is specified | 6 | 15.4 |
| Only the Outcome Discount Rate is specified | 1 | 2.6 |
| Neither Cost nor Outcome Rate is specified | 10 | 25.6 |
| Not applicable | 3 | 7.7 |
| **Types of costs** |  |  |
| Only Direct Costs | 31 | 79.5 |
| Direct and Indirect Costs | 8 | 20.5 |
| **Health Outcome Measure** |  |  |
| QALY gained | 19 | 48.7 |
| QALM gained | 2 | 5.1 |
| DALY averted | 3 | 7.7 |
| QALYs and Years of Life gained | 1 | 2.6 |
| Education costs averted (used as a measure of benefit in CBA) | 1 | 2.6 |
| Life Years gained | 2 | 5.1 |
| Life years gained and disability-free life years gained | 1 | 2.6 |
| Severe events avoided | 3 | 7.7 |
| Cases diagnosed | 4 | 10.3 |
| Surrogate clinical outcome | 1 | 2.6 |
| Composite measure of child physical function | 2 | 5.1 |
| **Incremental Analysis** |  |  |
| Yes, and ICER is reported | 32 | 82.1 |
| ICER not reported | 7 | 17.9 |

*CEA* cost-effectiveness analysis, *CEA* cost-benefit analysis, *CUA* cost-utility analysis, *QALY* quality-adjusted life-year, *QALY* quality-adjusted life-months, DALY disability -adjusted Life Year, ICER incremental cost-effectiveness ratio.
